# Eating Disorders in Taekwondo Athletes: The Contribution of Coach Behavior, Body Satisfaction, and Goal Orientation

**DOI:** 10.3390/sports12120315

**Published:** 2024-11-21

**Authors:** Renata Barić, Tanja Erdeljac

**Affiliations:** 1 Faculty of Kinesiology, University of Zagreb, 10000 Zagreb, Croatia; 2 MEDILAB, 21000 Split, Croatia; tanja.erdeljac@medilabone.com

**Keywords:** combat sport, body weight, mental health, mental disorder, elite athletes

## Abstract

Taekwondo is a weight-classified combat sport. Taekwondo athletes often resort to restrictive eating behaviors and weight reduction, especially just before competitions, which, in the long run, endanger their physical and mental health. This study aimed to determine the incidence of eating disorder (ED) symptoms regarding sex, age, and competitive level categories and examine the correlation and contribution of coach behavior, body satisfaction, and goal orientation to the development of ED symptoms in different subgroups. A total of 335 active Croatian taekwondo athletes, with a mean age of 14.7 years, among whom were 132 males and 203 females (range 10–26 yrs, *SD* = 3.06), completed the Croatian version of the Eating Attitude Test, Figure Rating Scale, Croatian Task and Ego Orientation in Sport Questionnaire, Negative Coach Behavior Questionnaire, and a questionnaire related to taekwondo practice. The results showed that ED symptoms were more present in female than male athletes (*p* < 0.001), while there was no statistically significant difference among the age and competitive level categories between elite and non-elite athletes. The risk of ED was significantly correlated (*p* < 0.001) with taekwondo athletes’ body dissatisfaction, coach pressure, and task goal orientation. In general, regression analyses showed that athletes’ body image dissatisfaction (*β* = 0.310; *p* < 0.001), coach pressure on diet and weight (*β* = 0.156; *p* < 0.005), and athletes’ task goal orientation (*β* = 0.120; *p* < 0.032) are statistically significant predictors that explain the variance in ED symptoms in Croatian taekwondo athletes. The culture of taekwondo sports represents a risk factor for ED development.

## 1. Introduction

Besides the positive contribution of regular exercise and sports to health in general, there is also a negative influence of competitive sport, especially to mental health [1,2], which is often considered taboo. The most common mental health issues related to sports are anxiety and depression symptoms, eating disorders (EDs), and disordered eating (DE) habits, all related to body dissatisfaction and the imperative need to achieve sports results [1]. In order to optimize sports performance, competitive athletes adhere to rigid diets and eating regimens that make them uniquely vulnerable to DE. Many of them use very strict methods of weight control, and there are sports with a higher risk for the development of EDs and DE, e.g., aesthetic sports and endurance and combat sports with strict demands of weight categories [3,4]. Scientific evidence shows that up to 45% of female athletes and up to 32.5% of male athletes have serious symptoms of DE or EDs [5]. Despite the multifactorial etiology of EDs, sports represents one of the strongest risk factors for the development of these disorders, which are more frequent, severe, and resistant in athletes than in non-athletes [3,6,7]. Taekwondo is a very popular Olympic combat sport, where having a lean and tall body with long limbs is considered desirable. The culture of taekwondo is marked with qualifying for lower weight categories in order to earn a competitive advantage [7,8]. Many taekwondo athletes use certain methods, e.g., chronic and acute manipulation of body weight, that can endanger their physical and mental health and sports performance [9]. Chronic body mass manipulation implies a continuous decrease in BMI during the months or weeks before a competition; acute manipulation implies restriction during the week or few days before a competition realized by dehydration, starvation, lowering glycogen levels, excessive exercising, salt baths, etc. [10], resulting in a 3–10% average reduction in athletes’ body mass. The more aggressive the body mass reduction method is, the higher the risk of health impairment. Repeating these procedures each competitive season represents a significant risk factor for ED development and also for different psychological difficulties. This can be related to self-esteem, self-confidence, a distorted relationship towards one’s body [11,12,13,14], or cognitive changes and concentration problems due to a low energy intake [15]. It also causes gastrointestinal, hormonal, and cardiovascular problems, as well as bone structure changes and female athlete triad, etc., that impair the training process and deteriorate sports performance, objectively and psychologically [16,17]. EDs most often begin in adolescence, when most athletes become more serious competitors [18]. During this period, the body biologically starts to change and grow. A socially imposed ideal of thinness is engendered, and it is especially difficult for female athletes to maintain a desired BMI [19]. Female adolescent athletes become more sensitive to the opinion of others and dissatisfied with their changes in body shape and size [20], especially if they are criticized or commented on by coaches or significant persons within the sports environment. Athletes of both sexes experience a strong socio-cultural pressure that accentuates a lean, athletic body as an ideal one. Also, certain desirable sports traits such as perfectionism, strict self-discipline, competitiveness, high ego and goal outcome orientation, worries about mistakes, etc., correlate to ED development [21,22,23]. EDs in sports appear as anorexia nervosa, often called anorexia athletica, but also as bulimia, excessive/binge eating, orthorexia, and different forms of non-specific DE behaviors.

How much a sport will contribute to EDs and DE depends on athletes’ dispositional variables and personal factors, e.g., age, personality traits, or motivational orientation, but also on situational factors such as the type and quality level of the sport or a coach’s behavior [14]. A coach’s leadership behavior directly impacts an athlete’s emotional reactions to his/her sport performance and influences the coach–athlete relationship as a framework of the athlete’s psychological development and motivation [24,25]. Inappropriate methods and toxic behavior can lead to low self-esteem, competitive anxiety, burnout, depression, isolation, negative body image, low self-efficacy, and the need to withdraw from sports [26,27]. Toxic coaches are highly demanding, judgmental, unsupportive, insufficiently instructive, and mainly autocratic leaders [28]. They use anger and negative comments about an athlete’s effort investment, performance, or body appearance as motivational tools, mainly unaware that they can lead to serious psychological difficulties such as EDs [28]. In taekwondo, coaches often overlook problems related to their athletes’ malnutrition, despite being constantly engaged in controlling their body mass [29]. On the contrary, coaches’ advice and demands for body mass reduction reinforce DE patterns [30]. They are the most significant factor that contributes to ED development, followed by negative comments from other sports personnel (assistant coaches and referees) about the necessity of weight loss, public weighting, and the need for food restriction to satisfy the limits of a lower weight category [31]. Also, one-third of coaches deny the severity of ED symptoms, trying to help athletes without any consultation with experts, psychologists, or nutritionists [32], which usually aggravates the issue. A coach’s praise for successful weight loss is one of the strongest mechanisms of maintaining EDs in combat sport athletes [33,34]. Athletes reported high pressure to satisfy their coach’s expectations and being afraid of losing their team position if they fail or disappoint their coach [33]. An unsatisfactory coach–athlete relationship can also contribute to ED development indirectly through the athlete’s self-criticism, internalization of their coach’s rules related to eating, low self-esteem, and anxiety and depression symptoms [35].

A personal factor that significantly contributes to EDs is body image dissatisfaction. This is a negative subjective evaluation of one’s own body as a whole or of specific aspects of the body, such as height, weight, shape, muscularity, or body fat [36]. It represents a discrepancy between the actual, self-perceived body image and the ideal, desired body image derived from comparing oneself to others. This discrepancy creates a negative affective response [37]—especially in those who place high importance on physique and appearance, as athletes usually do—and is especially due to the specific norms of certain sports. Body image dissatisfaction is emphasized in the current technological era where social networking enables continuous comparison worldwide, practically to everybody. This puts a large amount of pressure on youths, and social media has a stronger influence on young people’s body image aspirations and satisfaction than all other social influences [38]. This is especially true for athletes, as a specific population whose basic role depends on an effective and attractive body, which is evaluated in terms of time, speed, points, visual appearance, artistry, technical performance presentation, etc., as a basis for sports-related results. Previous research shows that women are more dissatisfied with their body image then men [39,40]. In sports, this exists in men too, especially in recent years [41], where there has been an increasing ED prevalence in male athletes [42]. Body image dissatisfaction among athletes can lead to different compulsive, excessive, and unhealthy types of behavior, including DE patterns such as starvation, dehydration, vomiting, laxative and diuretic use, and excessive exercising, in order to alter their physical appearance and compensate for anxiety or unpleasant emotions [43,44,45]. Maintaining the required body shape helps young taekwondo athletes gain positive feedback. It contributes to their self-confidence, which increases with successful attempts to reduce body mass [46] and reinforces further weight loss. Young athletes with a competitive mindset gradually start to compete with the pointer on the scale, experiencing a “dopamine rush” each time it goes down, which feeds motivation. According to goal orientation theory [47,48], athletes evaluate their success and sporting competence according to two different goals, namely, task goal orientation and ego goal orientation, which determine athletes’ choices, beliefs, and behaviors in the context of sports. Task-oriented athletes are primarily motivated by learning, personal improvement and mastery, and using self-referenced criteria to evaluate their achievements, and for them, to be successful means to be better than before [49,50,51,52]. They are intrinsically motivated [53], choose challenging goals, appreciate feedback [54], take responsibility for any sporting outcome, feel more optimistic, and show more positive attitudes toward themselves. An ego-goal-oriented athlete is primarily motivated by surpassing others and needs to demonstrate superior abilities and to be the best. Their success criteria are normative, based on constant comparison to other athletes [54]. This type of athlete believes that talent is the most important factor for sporting success [55], and their motivation depends on results and external recognition. Talent can be a strong source of pressure, and these athletes are ready to take all necessary measures to achieve the desired advantage and results and to win. De Bruin et al. [22], studying a sample of young female gymnasts and dancers with ED, found that ego goal orientation correlates with more pronounced dieting habits, higher peer pressure related to body mass, higher perfectionism, and lower self-esteem, leading to DE behaviors.

Competitive athletes operate within a strict sporting system where results and winning are dominant values, and the main criterion is success or failure. Most of them are oriented toward excellence and result-based achievements, and the same is expected from their social environment, which comprises coaches, parents, the media, and/or peers. It is a strong source of external pressure, which is compounded by internal ones [56,57]. To reach the highest goals, athletes are ready to sacrifice everything, even endangering their physical and mental health, for the silent approval of others. It is necessary to research and document such phenomena in sports in order to raise the awareness and understanding of the problem and to create evidence-based strategies for the prevention of EDs in sports.

This study had two aims: (1) to examine the presence of ED symptoms regarding sex, age, and competitive level categories and (2) to examine the correlation and contribution of coach behavior, body satisfaction, and goal orientation to the development of ED symptoms in different subgroups. We hypothesized that there are significant differences in the incidence of ED symptoms: we expected most ED symptoms to appear in the female and junior (14–17 yrs) categories as well as in elite taekwondo athletes. We also hypothesized that taekwondo athletes who perceive their coach’s behavior as more negative and pressuring in terms of eating and body weight, who are less satisfied with their body image, and who are more ego-goal-oriented will have more prominent ED symptoms. We expected that these variables would make a significant contribution to explaining the variance in ED symptoms in female and junior-age taekwondo athletes.

## 2. Materials and Methods

### 2.1. Participants and Procedure

In total, 335 active Croatian taekwondo athletes from 20 clubs in 7 different cities participated in this study. The inclusion criteria were active practice of taekwondo for at least 6 months and being a competitor in one of three competitive categories: cadets, junior athletes, or senior athletes. Athletes voluntarily participated in this study; those under the age of 14 participated after their tutors signed an informed consent form. The sample consisted of taekwondo athletes aged 10 to 26 years (14.01 ± 3.06), of whom there were 132 male participants (39.4%) and 203 female participants (60.6%). There were 175 cadets (52.2%), 98 junior athletes (29.3%), and 62 senior (18.5%) athletes. Among the participants, there were 58 (17.3%) top elite athletes, members of national selection, and elite athletes officially classed in the 1st, 2nd, or 3rd category by the Croatian Olympic Committee. Data collection was announced and approved by each club; it was carried out by two researchers, one of whom is an author of this article. It took place in February and March 2020, using paper and pencil, before or after a training session and lasted for about 25 min. The participants could withdraw from this study at any time and, because of the sensitivity of the investigation topic, a specially educated experimenter, a researcher for this study, was made available for the participants to talk to and debriefed the participants following the protocol that was prepared in advance, as necessary. The implementation of this research was approved by the Committee for Scientific Work and Ethics of the Faculty of Kinesiology, University of Zagreb (79/2020), and it was conducted in accordance with the local legislation, institutional requirements, and ethical considerations of scientific studies on humans.

### 2.2. Measures

#### 2.2.1. Sociodemographic Data, Sporting Experience, and Body Composition

The participants provided information on sociodemographic data, which included questions about age, sex, body height and weight, sporting experience and questions about age and weight categories and about belonging to the sports category determined by the Croatian Olympic Committee. There were also questions about experience and participation in the highest-level competitions (city championship, county or regional competitions, state championship, international club competitions, European championship, world championship, and Olympic games), and the participants were asked to self-assess their sporting success on a 1–5 scale (1—not at all, 5—extremely).

#### 2.2.2. Eating Disorder Symptoms

ED and DE risk was assessed using the Croatian version [58] of the Eating Attitudes Test (EAT-26) [59]. In this test, behaviors and habits related to nutrition are assessed using 26 items on a 6-point Likert scale (1—never, 6—always), and the total result is obtained by adding up the points on all items, with the answers that tend in the direction of pathological experiences or nonadaptive behavior receiving the most points. An answer that points to the greatest expression of symptoms of an ED (“6”) receives 3 points, an answer of “5” receives 2 points, and an answer of “4” receives 1 point. The answers “sometimes”, “rarely” and “never” are not scored. The theoretical score range is from 0 to 78. A cutoff score of 20 is considered critical, representing clinically indicative DE attitudes and behaviors [59]. Although this instrument allows the estimation of three dimensions, for the purposes of this study, to examine the presence of ED symptoms in accordance with the stated criteria, the total score was used and the instrument’s reliability was confirmed (Cronbach’s α = 0.74), which is congruent with previous applications of this instrument [60].

#### 2.2.3. Coach’s Behavior

An athlete’s evaluation of their coach’s behavior was obtained with the Coach’s Negative Behavior Questionnaire [61]. It contains 13 items divided into three subscales: insensitivity to the athlete’s personal well-being (“My coach doesn’t recognize athletes’ needs”), negative feedback (“My coach insults athletes on training sessions”), and focus on results (“My coach emphasizes the importance of winning”). Athletes estimate the frequency of their coach’s behaviors described by each statement on a 5-point Likert scale (1—never, 5—always). The mean score is calculated on each subscale and for the total score as an indicator of a coach’s negative behavior; a higher result indicates a higher frequency of a certain form of undesirable behavior from the coach. Cronbach’s alpha coefficients show adequate reliability in this study (insensitivity α = 0.76, negative feedback α = 0.83, focus on results α = 0.75, total score α = 0.80), which is congruent with previous investigations where Cronbach’s alpha coefficients ranged from 0.81 to 0.87 and 0.90 for the total score [61].

#### 2.2.4. Taekwondo Experience

Athletes also filled out a two-part questionnaire designed for this study, containing questions specifically related to taekwondo. The first part of the questionnaire consists of seven questions related to personal attitudes and beliefs concerning the reduction in body weight in taekwondo (e.g., “I am not tall enough for my weight category and I would be more competitive in a lower weight category”). The participants assessed the degree of agreement with each statement on a scale from 1 to 5 (1—strongly disagree; 5—strongly agree), and the results were presented as a percentage for each question; Cronbach’s α = 0.52. The second part consists of nine questions and serves to assess the frequency of some coaches’ behaviors (e.g., “My coach makes jokes about my appearance and my body weight”) on a 5-point scale (1—never; 5—always). The results were expressed as the average value of all answers and represented an index of the pressure placed on athletes by taekwondo coaches in relation to nutrition and body weight; Cronbach’s α = 0.62. This instrument was constructed for the purpose of this research, based on the authors’ long-term experiences in the sports field, namely, experiences in elite taekwondo, taekwondo parenting, and sports psychology. The athletes’ ability to understand the content was checked on a small test sample of taekwondo athletes, prior to the data collection procedure.

#### 2.2.5. Body Satisfaction

Satisfaction with body image was estimated using the Figure Rating Scale (FRS) [62], a widely used instrument in studies of body image in athletes [40,63]. The Stunkard scale consists of nine male and female schematic figures ranging from very thin to obese. The participants were asked to make two assessments: on the first row of figures, to select the figure that most closely represents their current body image, and, on the row below, to choose the figure that represents their ideal body image. The difference between the two represents the measure of individual body image dissatisfaction. The theoretically possible range of results is between −8 and 8. The larger the absolute difference, the less satisfied the person is with their body, while the sign indicates the direction of dissatisfaction (the minus sign suggests that the person wants to increase body mass; the plus sign indicates that the person wants to reduce body mass).

#### 2.2.6. Motivation

**Figure 1:**
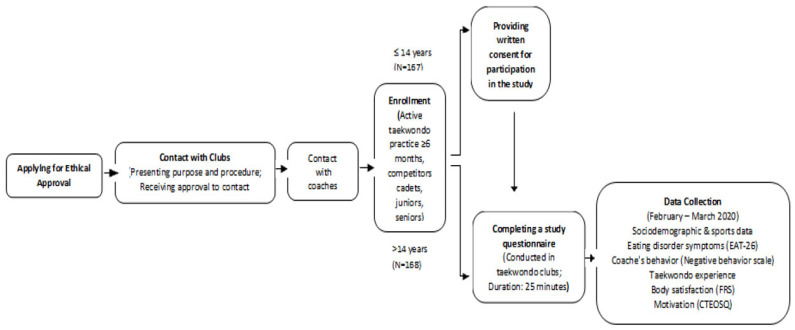
Flowchart diagram of this study.

Athletes’ dispositional motivational orientation was assessed with the Croatian Task and Ego Orientation in Sport Questionnaire (CTEOSQ) [64,65]. The questionnaire comprises two composite scales: a 7-item task subscale, (“I learn a new skill and it makes me want to practice more”) and a 6-item ego subscale (“I can do better than my friends”). The stem “I feel most successful in taekwondo when…” preceded each item. The Cronbach’s alpha coefficients showed the good reliability of this questionnaire in the current study (task α = 0.86, ego α = 0.82) as is the case in previous ones [64,66]. The participants responded using a 5-point Likert-type scale (1—strongly disagree; 5—strongly agree). Athletes were asked to respond to the CTEOSQ regarding their participation in training sessions and competitions within the current season. The study design is presented in Figure 1.

### 2.3. Statistics

The Kolmogorov–Smirnov test was used to check the normality of all the measures. The Mann–Whitney test was used to compare the incidence of ED symptomatology between the sexes and level-of-competition subgroups, while the Kruskal–Wallis test was used to compare that between age categories. Descriptive parameters of demographic data and all of the variables and Spearman’s correlation coefficients were calculated. To determine the contribution of coach behavior, body satisfaction, and goal orientation to ED symptoms, multiple regression analyses were performed separately for different sexes, competitive age categories, and level-of-competition subsamples. Because the results of the KS test indicated the non-normality of the distribution of results, an analysis of residuals was conducted, and a normal probability plot was constructed [67]. These analyses showed that the variables used in this research exhibit multivariate normal distribution, which enables the use of multiple regression analyses. A significance level of *p* < 0.05 was established for all the analyses. Finally, to better understand the background of restrictive dieting for competitive advantage in taekwondo and the possible background of EDs in taekwondo athletes, a descriptive analysis was carried out using the percentage of responses of athletes’ attitudes and beliefs about diet, body weight reduction, and current conditions related to it. Statistical analyses were conducted using IBM SPSS Statistics for Windows version 29, Premium Campus Edition, SPSS ID: 729030.

## 3. Results

### 3.1. Descriptive Analysis and Prerequisites for Using Parametric Statistical Procedures

Before selecting statistical procedures for data processing, the conditions for using parametric tests were assessed. The Kolmogorov–Smirnov test was used to check the normality of the variable distributions. It was found that the results of all the eight variables deviated in a statistically significant manner from the normal distribution (*p* < 0.05). Skewness coefficients ranged between 0.20 (ego goal orientation) and 2.50 (coach pressure), grouping in the zone of higher results, with the exception of task goal orientation (skew = −1.78). Kurtosis coefficients ranged between −0.41 (ego goal orientation) and 11.71 (coach pressure). Therefore, non-parametric tests were used for processing. The average body weight was 52.00 ± 12.61 kg, ranging from 25.00 to 96.00 kg. The average height of the participants was 1.65 ± 0.11 m, ranging from 1.32 to 2.05 m. The average body mass index (BMI) was 19.25 ± 2.82 kg/m^2^, and the BMI ranged from 13.59 to 32.08 kg/m^2^.

### 3.2. Symptomatology of Eating Disorders

To examine the presence of ED risk based on attitudes, feelings, and behaviors related to eating with regard to sex, age, and competitive level categories, descriptive statistics for each subgroup were produced (Table 1 and Table 2), and the differences between subsamples were examined using Mann–Whitney and Kruskal–Wallis tests.

Taking into account the cutoff value of 20 [56], these results show that 15.3% of female and 2.3% of the male taekwondo athletes show a high level of concern about nutrition and body weight; i.e., ED and DE symptoms are present in 9.2% of the Croatian taekwondo athletes of this sample. A higher prominence of symptoms is present in younger than in older categories, primarily in cadet and junior girls (who have the highest mean score on the EAT-26 test), and the range of scores from 1 to 53 in the female and 0 to 33 in the male taekwondo athletes show that some of them suffer from severe ED symptoms (Table 1 and Table 2). This finding is mainly in line with BMI values, as another criterion for EDs. On average, the female taekwondo athletes have a BMI of 19.05 and the male taekwondo athletes have a BMI of 19.56, which correspond to low–normal weight [65]. Considering age and BMI, the average cadet and junior taekwondo athlete belongs to the normal or low–normal weight category. BMI is not a reliable criterion for determining EDs, especially in children and youths, as it depends on age, sex, and body type, and it is often the case that athletes have a normal BMI due to their muscular bodies but serious ED symptoms. Within the cadet and junior competitive category, there are two 3-year ranges (cadets: 12–14; junior athletes: 15–17 yrs), and each age range has a slightly different BMI value, which is critical for underweight according to norms obtained from Croatian samples [68]. We decided to take the BMI value for the middle age of the category as the representative one (Table 1). The data show that more athletes from younger categories have a BMI below the limit that corresponds to underweight or severe underweight [68] (Table 1). On the other hand, the data show that two cadet and three junior female athletes and four cadet, one junior, and one senior male athlete are overweight (BMI ≥ 25), and two of them are obese (BMI ≥ 30) [69].

The results presented in Table 2 show that the motivational tendencies of all the athletes across all the subsamples are characterized by a higher level of task than ego goal orientation. Both the male and female taekwondo athletes are quite satisfied with their physical appearance; the male athletes have a slight desire to increase (M = −0.17 ± 1.26) and the female athletes have a slight desire to decrease (M = 0.39± 0.93) their body weight. The junior athletes are less satisfied with their physical appearance than the cadets and senior athletes. The elite athletes are completely satisfied with their physical appearance. Both the male and female taekwondo athletes similarly experience negative behavior and pressure from their coaches. All the age categories of the athletes do not consider their coaches’ behavior as very negative, mostly perceiving their coaches as focused primarily on results. The senior athletes and elite taekwondo athletes perceive the highest level of pressure from their coaches in relation to nutrition and weight (M = 1.87 ± 0.87).

To compare the incidence of ED and DE symptoms between subsamples, the Mann–Whitney (sex and competitive quality level) and Kruskal–Wallis (competitive age categories) tests were applied. A statistically significant difference in the occurrence of ED symptoms was determined regarding gender (*Z* = –4.604; *p* < 0.001). Symptoms of ED and DE, as expected, occur more often in the female (*Mf* = 11.16; *SD* = 7.87) than in the male taekwondo athletes (*Mm* = 6.00; *SD* = 5.11). While 15.27% of the female taekwondo athletes have a critical score on the EAT-26, the same was obtained for 2.27% of the male taekwondo athletes (Table 1). In contrast to our initial hypothesis, a statistically significant difference in the occurrence of ED symptoms was not found between the elite and non-elite taekwondo athletes (*Z* = –0.028: *p* = 0.977). Symptoms of ED were found in 6.9% of the elite taekwondo athletes, and all critical results were recorded in the females, while 10.8% of the critical results were found in the non-elite athletes. No significant difference in the incidence of ED and DE symptoms was found among the age categories (*H* (2) = 0.999, *p* = 0.607). However, the trend of occurrence of symptoms of eating disorders is more often observed in the category of junior athletes (12.2%), followed by cadets (9.7%) and senior athletes (8.0%).

#### 3.2.1. Correlation and Contribution of Predictors to ED and DE Symptoms

To examine the connection between all variables used in this research, Spearman’s correlation coefficients were calculated (Table 3).

The results show that task goal orientation, body image dissatisfaction, and pressure in relation to nutrition from coaches are significantly and positively correlated with ED and DE symptoms. Taekwondo athletes who are more focused on learning, improvement, and personal development, who are less satisfied with their physical appearance (with a desire to lower their body mass), and who feel more pressure from the coach regarding diet and weight suffer from more ED symptoms. Other predictor variables are not significantly related to the prevalence of ED symptoms. Furthermore, other correlations show a logical relationship between the tested variables, for example, the index of pressure regarding nutrition from coaches is positively correlated with all the variables of a coach’s negative behavior, which indicates a leadership style that is risky for an athlete’s well-being. Correlations were tested with regard to sex (Table 4). There is a correlation between task goal orientation and increased coaches’ sensitivity and EDs in the male taekwondo athletes and between body image dissatisfaction and pressure regarding nutrition from coaches in the female taekwondo athletes, which provides a different context for this disorder.

To test whether there was a significant contribution of predictor variables to the development and expression of ED symptoms in athletes of different sex and age categories and competitive level categories, several regression analyses were performed (Table 5).

The results of the regression analyses (Table 5) show that the most significant predictive contribution to the explanation of the variance in ED symptoms is body image dissatisfaction. The next are pressure regarding diet and weight from coaches, task goal orientation, and insensitivity of coaches toward cadets and non-elite athletes, which partially confirmed our hypothesis. Ego goal orientation, coach’s negative feedback, and focus on results are not significant predictors for ED symptoms in any subgroup. In the female taekwondo athletes, 17% of the variance in ED symptoms was explained by body image dissatisfaction. In the male athletes, no single significant predictors were found, but taken together, this set of predictor variables statistically significantly explains 14% of the variance in ED symptoms. Furthermore, 14.1% of the variance in ED symptoms in the cadet taekwondo athletes was explained, mainly by the athletes’ dissatisfaction with their physical appearance and by the coach’s low insensitivity, i.e., the coach’s possible hypersensitivity or overcontrolling of athlete’s needs and well-being. In the sample of junior athletes, 24.6% of the variance in ED symptoms was explained primarily by the athletes’ dissatisfaction with their physical appearance and pressure regarding diet and weight from coaches. In the senior athletes, these predictors do not significantly explain the variance in the development of ED symptoms, although the predictor variables with the highest beta values are the same as in the subsamples of the younger categories. It is possible that such a finding is a consequence of the lower number of participants in the subsample. Although the results show a nonsignificant contribution, the same trend is obvious. However, this set of predictor variables significantly contributes to the development of ED and DE symptoms in the non-elite taekwondo subsample, explaining 17.8% of the variance. ED symptoms are best explained by body image dissatisfaction, then pressure regarding diet and weight from coaches, hypersensitivity of coaches to athletes’ needs, and athletes’ task goal orientation.

#### 3.2.2. Personal Attitudes and Beliefs About Weight and Eating Behavior in Croatian Taekwondo Athletes

To better understand the motivational background of restrictive diets aiming to increase the chances of achieving a better result in taekwondo, and to obtain additional insight into the possible origins of the development of ED and DE symptoms, it is considered important to examine how athletes generally view this issue concerning their sport and related factors. The participants reported their attitudes and beliefs related to the reduction in body weight in taekwondo, which represent the first part of the questionnaire designed for this research (Table 6).

It is evident that 44% of the participants think it is normal to reduce body weight for taekwondo competitions. This is in line with the data from about 32% of the Croatian taekwondo athletes who believe that they are not tall enough for their current competitive weight category and that they would have better chances of achieving results in a lower weight category. At the same time, 35% of the participants are afraid that they will lose strength if they reduce body weight, and 39% believe that the targeted reduction in body weight will have long-term consequences for their physical or mental health. Looking in detail at how the participants who scored a critical score (≥20 points) on the EAT-26 answered this question, we found that 32% of them believe that the targeted reduction in body weight will have long-term consequences for their physical or mental health, but despite this, they reduce their weight on purpose, for the competition. In total, 43% of the participants do not pay attention to diet at all or mostly in periods where there are no competitions, while 31% do, and 23% of the participants generally avoid weighing themselves. Based on one of the additional questions, “How often do you weigh yourself?”, 6.0% of the participants weigh themselves several times a day (an ED behavioral symptom), and 40.9% of the participants weigh themselves once a week. As they are the ones who put the most pressure on themselves regarding diet and body weight, the participants mostly mentioned themselves as the source of pressure (50.7%). Moreover, 23.0% of the participants feel such pressure from their parents, more from their mothers (18.5%) than their fathers (16.7%). In total, 6.6% of the taekwondo athletes reported that their coach represents the strongest source of social pressure related to body weight and nutrition, while 2.4% of the participants reported that the highest pressure related to diet and body weight is created by friends from their club and, for 0.6% of the athletes, from the national team coach.

## 4. Discussion

The aim of this research was to examine the prevalence of and differences in ED symptoms regarding sex, age, and competitive level in Croatian taekwondo athletes and to determine the correlation and contribution of coach behavior, body satisfaction, and goal orientation with the development of ED symptoms in different subgroups. The results show that 9.2% of the athletes have ED and DE symptoms, and 15.3% of the female and 2.3% of the male taekwondo athletes show a high level of concern about dieting and body weight and exhibit problematic eating behaviors, and this difference is statistically significant, which corresponds with our initial presumption. In previous findings, these percentages vary: Martinez-Rodriguez et al. [70] found that 2% of Spanish taekwondo and karate athletes of cadet age meet the criteria for ED diagnosis. These percentages went up to 45% in female and 35.5% in male athletes [71,72]. Gonçalves et al. [73] reported that about 64.4% of combat sport athletes with ED symptoms and behavior, such as fasting, intense physical activity, laxative misuse, binge eating, and induced vomiting, aimed to prevent weight gain or reduce weight prior to a competition. Regarding BMI, another indicator of possible ED risk, the results show that Croatian taekwondo athletes in general fall into the low–normal weight category: the average female taekwondo athlete has a BMI of 19.05, and the average male taekwondo athlete has a BMI of 19.56 [69]. Of this sample, 9% of the female cadet and junior taekwondo athletes, mainly girls, have a BMI that indicates they are underweight or severely underweight [69]. This can be an indicator of an ED, especially if associated with specific behavioral patterns (e.g., strict dieting and body weight management for competition purposes; starvation; and excessive exercising), as it is in combat sport athletes. This finding is congruent with previous research on sport that consistently confirms a higher prevalence of EDs in female athletes [5,71,74,75]. It can be explained with hormonal changes in puberty and early adolescence, which alter the body, especially in terms of weight increment, which is undesirable for taekwondo. Girls do not want to compete in a heavier weight category, so they intentionally lose weight. The lower incidence of EDs in male athletes could be related to a stronger stigma about mental disorders, especially in men in sport, and their resistance to speaking about it or seeking help, which probably leaves many men with undiagnosed EDs. Chapman and Woodman [75], in their meta-analysis of EDs in male athletes, raise concerns about methodological issues related to the diagnostic tool EAT-26. According to them, this scale is considered to be a better predictor of EDs in women because it was constructed with female symptoms in mind and involves concerns about body weight, shape, dieting, etc., while some better indicators of ED symptoms in men, such as muscle dysmorphia or binge eating, are missing [75]. Although the results show that most of the ED symptoms in taekwondo athletes reflect underweight and disturbances related to anorexia nervosa, there are a few young overweight (BMI > 25)—even obese—female and male athletes (BMI > 30) [69]. This corresponds to excessive eating difficulties as a possible consequence of long-term restrictions and strict dieting, which cause emotional problems and difficulties with self-control in relation to eating. Contemporary ED conceptualization presumes transdiagnostic theory [76], which considers EDs as unique problems with common background mechanisms, not as three discrete ones. A person with an ED moves along that continuum and back depending on the phase of the disorder or recovery. This can be a mechanism that helps explain different manifestations of EDs and transitions between symptoms, and it is often recognized among taekwondo athletes.

Initially, we also expected to find a higher risk of ED and DE development in junior athletes and in top elite athletes. The results showed a higher incidence of ED symptoms in younger than in older taekwondo athletes, primarily in cadets and junior girls, although these differences were not statistically significant. The obtained trends of results regarding competitive age categories show that junior athletes are at a slightly higher risk of ED development, which is in line with the developmental dynamics of EDs and DE, which are most pronounced in adolescence [60,77]. Contrary to our expectations, no significant difference in ED symptoms in elite taekwondo athletes in comparison to non-elite athletes was found, and this finding is in agreement with the results of Teixidor-Batlle and collaborators [74], who showed that competition level was not an additional risk factor for EDs in elite Spanish athletes beyond the sport itself. However, our results show a trend of higher risk for ED development in non-elite athletes. This can be explained by their drive toward sporting success that has not been reached yet and by their willingness to take all necessary measures to achieve it. Some studies have shown the opposite findings, stating that non-elite athletes have a reduced risk of developing an ED in comparison to elite ones [14]. Our hypothesis was based on the fact that elite athletes experience more intensive, more frequent, and more exhausting training as well as more frequent body weight regulation not only due to tight competitive schedules [78] but also higher social pressure from coaches, the media, and the public. It is possible that elite competitors have accepted the strict sporting lifestyle required to be a taekwondo competitor and have learned how to regulate their body mass in a healthier way.

The results also show that athletes’ task goal orientation, body image dissatisfaction, and pressure regarding dieting from coaches are significantly and positively correlated with ED and DE symptoms. In other words, taekwondo athletes who are more focused on progress and improvement, who are motivated by learning and more prepared to surpass their own limits to develop, who are ready to embrace hard and more challenging goals, and who are less satisfied with their physical appearance in a way that makes them want to lose weight and body fat are at greater risk of developing DE habits and EDs. Previous studies have shown a positive correlation between ego goal orientation and ED symptoms [22]; i.e., ego goal orientation was significantly correlated with different unhealthy dieting methods, with lower self-esteem, and higher perfectionism. The results obtained from 290 Portuguese athletes are congruent with ours; the authors reported a significant positive correlation between task goal orientation and EDs [79]. It is possible that underlying this relationship is a perfectionism trait that correlates with task orientation [80,81]. Athletes with a perfectionistic mindset leave nothing to chance so that their performance can be as perfect as possible, and to achieve this, they also need a perfect body.

Our results are in agreement with those of Whitehead et al. [82], who state that dissatisfaction with body image is one of the strongest predictors of EDs in female athletes. Kristjánsdóttir and collaborators [83] found a high positive correlation between body dissatisfaction and ED symptoms in both male and female elite Icelandic athletes, 755 from 20 different sports. In sports, a perfect body necessary for the best performance often does not coincide with a desirable or trendy body image, according to social standards. Athletes who do not reach the ideal body type for their sport experience more pressure to achieve it, especially because it is less risky from a result-driven perspective, and start to use exaggerated methods that can progressively lead to an ED. Gomes and collaborators [79] reported that about 51.4% of the athletes from their study expressed a desire to lose weight despite 82.2% of the sample having a normal body weight. A lean and tall body with a low BMI and low body fat is a desirable body shape in taekwondo [8,84]; both male and female athletes strive to achieve this. Accordingly, taekwondo athletes are a risk group for the development of EDs. Athletes are usually more vulnerable to general pressures to attain an ideal sport body shape and very often experience sport-specific pressures from their sporting environment. A significant level of such pressure related to athletes’ nutrition, food monitoring, weight loss, or body image can be induced by coaches, which is also a significant predictor of ED symptoms according to the results of this study. This finding is also congruent with previous studies [4,85,86], and pressure from coaches is considered one of the main risk factors for body image concerns and DE habits in athletes [87,88]. It is especially true for those athletes with weak relationships with their coaches because a good coach–athlete relationship may reduce the negative impact of perceived weight-related coach pressure on the development of DE and EDs. The results of this study show that coach pressure regarding dieting and body weight is significantly correlated with all aspects of a coach’s negative behavior/insensitivity to athletes’ needs, tendency to give negative feedback, and dominant orientation toward sporting results. Such behavior can indirectly influence the increment in ED risk, through self-criticism, low self-esteem, and social anxiety due to body image [28]. Coaches usually demand that their athletes be self-critical and perfectionistic, to aim for the highest goals, and avoid failure, which are typical characteristics of an ED mindset. Nonetheless, some evidence shows that a caring and supportive coach can be a protective factor for ED development in a strict sports environment [89,90].

## 5. Conclusions

We can conclude that goal orientation, body dissatisfaction, and negative behavior and pressure from coaches significantly contribute to the risk of ED development in female and male cadet, junior, and non-elite athletes, while body dissatisfaction seems to be the strongest predictor, especially in female athletes. Symptoms of EDs in cadets occur more often in taekwondo athletes whose coach is excessively sensitive and worried for their well-being and in those who are not satisfied with their physical appearance. In the junior age category, ED symptoms are more likely to be developed by taekwondo athletes who are dissatisfied with their physical appearance and those who are pressured by the coach in relation to diet and body weight. In the category of non-elite athletes, the risk of ED increases if the athlete is more task-oriented, less satisfied with their body image, more pressured by the coach about dieting and weight loss, and if athlete’s coach is oversensitive to the athlete’s needs and becomes too controlling. Athletes’ personal values and beliefs illustrate that the culture of taekwondo presents a risk of ED development due to the traditions, patterns, and behaviors that are transmitted to athletes directly, through requests and recommendations but also indirectly through social learning and social expectations. The results show that losing weight is a common practice in this sport that is practiced even though athletes believe that it can endanger their physical and mental health and sports performance, yet they adhere to the social norms. As Croatian taekwondo athletes have confirmed their world-class excellence in the last decade, the system is successful, although the approach of “the end justifies the means” endangers mental health. Such behaviors and self-evaluations gradually extend beyond sport, become a part of their everyday life, become their mindset, their need, and their obsession, and transform into an ED mindset, which is the core of this mental disorder. The later it is recognized as a disorder, the more difficult it is to treat.

The scientific contribution of this study is to offer new scientific evidence of EDs and DE in sports—namely, in one that presents the highest risk (taekwondo)—and to recognize different dispositional and situational factors that contribute most to the development of this mental disorder. This way, it is possible to increase awareness of the dark side of this sport and to identify the foundation for treatment, education, and prevention, especially considering the mass appeal of taekwondo as a sport worldwide. Moreover, this research covers an evident gap in ED research conducted on Eastern European athletes, especially elite ones. It is also one of the few studies to examine the contribution of goal orientation to EDs in athletes.

One of the limitations of this study is the sample size, although it is realistic in relation to the number of active taekwondo competitors in Croatia. Future research should feature an increased sample size by including other combat sport athletes, as well as athletes from other sports who are at risk of ED development. To identify more male athletes with ED issues, we recommend including other measures, more adjusted to male ED symptoms. As the results showed the significance of the behavior of coaches in relation to athletes’ ED symptoms, it would be very interesting to investigate the transgenerational transfer of distorted values, beliefs, and behaviors related to EDs from coaches to athletes, i.e., to investigate coaches’ history of ED symptoms and their current behavior. Moreover, we used self-reported questionnaires. Due to the sensitivity of the topic, it is possible that the participants were not truthful when answering the EAT-26 or that some participants, especially the younger ones, could have misunderstood some or part of the questions or presented bias in their answers. We recommend conducting one-to-one interviews in future studies to obtain more reliable information.

## Figures and Tables

**Table 1:** Prevalence of ED symptoms and BMI values within the sample.

	N	EAT26 ≥ 20	EAT26 Score Range	Critical BMI Value	
Cadet	M: 69 F: 106	3 (4.35%) 14 (13.2%)	1–33 1–53	BMI < 15	3 (4.35%)
				10 (9.44%)	
Junior	M: 34 F: 64	12 (18.8%) 0 (0%)	1–19 1–34	BMI < 16.3 BMI < 16.5	3 (8.82%) 3 (4.69%)
Senior	M: 29 F: 33	0 (0%) 5 (15.2%)	0–16 2–35	BMI < 17.2 BMI < 17	0 (0.00%) 1 (3.03%)
Elite	M: 25 F: 33	0 (0%) 4 (12.1%)	0–16 1–35		
Non-elite	M: 107 F: 170	3 (2.8%) 27 (15.9%)	1–33 1–53		
Total	M: 132 F: 203	3 (2.27%) 31 (15.3%)	0–33 1–53		

Legend: M—male, F—female; BMI is not specified for mixed age categories.

**Table 2:** Descriptive statistics regarding sex (Nf = 203, Nm = 132), age category (Nc = 175, Nj = 98, Ns = 62), and competitive quality level (Ne = 58, Nn-e = 277).

Subsamples		Age	Weight	Height	BMI	EAT	Task	Ego	BID	INS	NF	RO	CP
MALE	M SD	14.9 3.40	56.99 14.29	170.0 13.10	19.56 2.90	7.43 * 5.11	4.12 0.78	2.87 0.95	−0.17 1.26	1.75 0.71	1.32 0.48	2.77 0.93	1.55 0.67
FEMALE	M SD	14.6 2.81	51.69 10.93	165.0 8.90	19.05 2.76	11.16 * 7.87	4.36 0.60	2.65 1.10	0.39 0.93	1.72 0.70	1.31 0.60	2.42 0.93	1.58 0.58
CADET	M SD	12.6 0.94	46.51 9.82	159.0 8.00	18.17 2.70	9.53 7.34	4.33 0.68	2.62 1.04	0.20 0.90	1.65 0.68	1.29 0.63	2.52 0.99	1.43 0.46
JUNIOR	M SD	15.5 0.83	59.19 10.26	171.0 8.00	20.17 2.76	10.28 7.11	4.10 0.76	2.68 1.02	0.67 1.42	1.82 0.72	1.30 0.47	2.56 0.90	1.63 0.59
SENIOR	M SD	19.9 2.51	65.74 9.20	171.0 7.70	20.17 2.76	9.23 6.67	4.36 0.57	3.16 1.02	−0.08 1.08	1.86 0.74	1.38 0.45	2.67 0.87	1.87 0.87
ELITE	M SD	18.4 3.48	63.44 10.74	175.0 8.54	20.49 2.20	9.38 6.62	4.39 0.49	3.03 0.97	0.00 1.12	1.68 0.68	1.31 0.40	2.57 0.83	1.75 0.77
NON-ELITE	M SD	14.0 2.35	51.75 12.04	164.0 10.55	18.99 2.87	9.75 7.25	4.24 0.72	2.67 1.06	0.20 1.10	1.75 0.71	1.31 0.59	2.58 0.97	1.53 0.57

Legend: Age in years, weight in kg, and height in cm. BMI—body mass index, EAT—the score based on the EAT-26 questionnaire, Task—task goal orientation, Ego—ego goal orientation, BDI—body dissatisfaction index, INS—coach’s insensitivity, NF—coach’s negative feedback, RO—coach’s orientation toward result achievement, CP—pressure from coach in relation to diet and body weight, * significant difference.

**Table 3:** Spearman’s correlation coefficients between variables (N = 335).

	1.	2.	3.	4.	5.	6.	7.
1. EAT-26 (ED symptoms)							
2. Ego goal orientation	−0.028						
3. Task goal orientation	0.16 **	0.12 *					
4. Body image dissatisfaction	0.27 **	−0.15 **	−0.08				
5. Insensitivity to athletes’ well-being	−0.11	0.01	−0.37 **	0.08			
6. Coach’s negative feedback	−0.01	0.07	−0.25 **	0.02	0.36 **		
7. Coach’s focus on results	0.01	0.15 *	−0.04	0.047	0.15 **	0.33 **	
8. Coach’s pressure in relation to nutrition	0.17 **	0.01	−0.16 **	0.13 *	0.18 **	0.38 **	0.32 **

** *p* < 0.01; * *p* < 0.05.

**Table 4:** Spearman’s correlation coefficients between variables (Nf = 203, Nm = 132).

	Male	Female
	EAT 26	
Ego goal orientation	−0.11	0.40
Task goal orientation	0.19 *	0.07
Body image dissatisfaction	0.05	0.31 **
Insensitivity to athletes’ well-being	−0.21 *	−0.04
Coach’s negative feedback	−0.01	0.03
Coach’s focus on results	0.05	0.07
Coach’s pressure due to nutrition	0.14	0.17 **

** *p* < 0.01; * *p* < 0.05.

**Table 5:** Contribution of predictor variables to the development of ED symptoms in different subsamples of Croatian taekwondo athletes (β).

	Female N = 203	Male N = 132	Cadet N = 175	Junior N = 98	Senior N = 62	Elite N = 58	Non-Elite N = 277	Total *N = 335*
Task goal orientation	0.102	0.084	0.144	0.089	0.233	0.140	0.124 *	0.120 *
Body image dissatisfaction	0.356 **	0.149	0.272 **	0.375 **	0.160	0.086	0.334 **	0.310 **
Insensitivity to athletes’ well-being	−0.060	−0.0185	−0.188 *	0.053	0.123	0.275	−0.127 *	−0.068
Coach’s pressure due to nutrition	0.142	0.168	0.064	0.222 *	0.228	0.193	0.157 *	0.156 *
* < 0.05 ** < 0.001	R = 0.413 R^2^ = 0.170 F = 5.715 **	R = 0.374 R^2^ = 0.140 F = 2.875 **	R = 0.376 R^2^ = 0.141 F = 3.930 **	R = 0.496 R^2^ = 0.246 F = 4.206 **	R = 0.456 R^2^ = 0.205 F = 1.992	R = 0.390 R^2^ = 0.152 F = 1.279	R = 0.442 R^2^ = 0.178 F = 8.349 **	R = 0.388 R^2^ = 0.150 F = 8.258 **

**Table 6:** The percentage of answers on personal attitudes and beliefs about weight and eating behavior in Croatian taekwondo athletes (N = 335).

	Strongly Disagree	Mainly Disagree	I’m Not Sure	Mainly Agree	Strongly Agree
I believe that, in taekwondo, it is normal to reduce body weight. If everyone is losing weight, why shouldn’t I?	14%	16%	26%	27%	17%
I am not tall enough for my weight category and would be more competitive in a lower weight category.	37%	13%	18%	16%	16%
I am afraid that I will lose strength if I reduce my body weight.	26%	20%	19%	20%	15%
I believe that a reduction in body weight will not have long-term consequences for my physical health.	21%	18%	30%	19%	12%
I believe that a reduction in body weight will not have long-term consequences for my mental health.	21%	18%	26%	16%	19%
Even when I don’t have a competition, I pay strict attention to my diet.	21%	22%	26%	24%	7%
I avoid weighing myself.	45%	17%	15%	12%	11%

## Data Availability

Researchers who wish to obtain the data used in this study can contact Professor Renata Barić, Ph.D.
